# Supplementary material for: A mapping review of methicillin-resistant Staphylococcus aureus proportions, genetic diversity, and antimicrobial resistance patterns in Cameroon
Source: PLoS One. 2023 Dec 22;18(12):e0296267. doi: 10.1371/journal.pone.0296267 (PMC10745167; doi:10.1371/journal.pone.0296267)
Supplement: S2 Table — (DOCX) [file pone.0296267.s002.docx]

S2 Table: Search strategy

| **Database** |  | Search | Items |
| --- | --- | --- | --- |
| **Medline (Ovid)** | 1 | exp Methicillin-Resistant Staphylococcus aureus/ | 19071 |
|  | 2 | Methicillin-Resistant Staphylococcus aureus.mp. | 29298 |
|  | 3 | MRSA.mp. | 24151 |
|  | 4 | exp Cameroon/ | 6443 |
|  | 5 | (Cameroon or Yaounde or Bertoua or Ebolowa or Maroua or Garoua or Ngaoundere or Bamenda or Buea or Bafoussam or Douala).mp. | 8252 |
|  | 6 | 1 or 2 or 3 | 34385 |
|  | 7 | 4 or 5 | 8252 |
|  | 8 | 6 and 7 | 16 |
|  |  |  |  |
| **Embase (Ovid)** | 1 | exp methicillin resistant Staphylococcus aureus/ | 55392 |
|  | 2 | methicillin resistant Staphylococcus aureus.mp. | 67253 |
|  | 3 | MRSA.mp. | 42312 |
|  | 4 | exp Cameroon/ | 8647 |
|  | 5 | (Cameroon or Yaounde or Bertoua or Ebolowa or Maroua or Garoua or Ngaoundere or Bamenda or Buea or Bafoussam or Douala).mp. | 11323 |
|  | 6 | 1 or 2 or 3 | 73186 |
|  | 7 | 4 or 5 | 11323 |
|  | 8 | 6 and 7 | 23 |
|  |  |  |  |
| **Global Health (Ovid)** | 1 | Methicillin-Resistant Staphylococcus aureus.mp. | 21806 |
|  | 2 | methicillin resistant Staphylococcus aureus.mp. | 21806 |
|  | 3 | MRSA.mp. | 21837 |
|  | 4 | (Cameroon or Yaounde or Bertoua or Ebolowa or Maroua or Garoua or Ngaoundere or Bamenda or Buea or Bafoussam or Douala).mp. | 9321 |
|  | 5 | 1 or 2 or 3 | 22140 |
|  | 6 | 4 and 5 | 20 |
|  |  |  |  |
| **Web of Science** | 1 | All fields = (Methicillin-Resistant Staphylococcus aureus OR methicillin resistant Staphylococcus aureus OR MRSA) AND (Cameroon OR Yaounde OR Bertoua OR Ebolowa OR Maroua OR Garoua OR Ngaoundere OR Bamenda OR Buea OR Bafoussam OR Douala) | 40 |
|  |  |  |  |
| **African Index Medicus** | 1 | Methicillin-Resistant Staphylococcus aureus OR methicillin resistant Staphylococcus aureus OR MRSA | 10 |
|  |  |  |  |
| **African Journal Online** | 1 | (Methicillin-Resistant Staphylococcus aureus OR methicillin resistant Staphylococcus aureus OR MRSA) AND (Cameroon) | 31 |
