# Supplementary material for: A mapping review of methicillin-resistant Staphylococcus aureus proportions, genetic diversity, and antimicrobial resistance patterns in Cameroon
Source: PLoS One. 2023 Dec 22;18(12):e0296267. doi: 10.1371/journal.pone.0296267 (PMC10745167; doi:10.1371/journal.pone.0296267)
Supplement: S3 Table — (DOCX) [file pone.0296267.s003.docx]

S3 Table: Items for risk of bias assessment

|  | Yes (1) | No (0) | Unclear (0) | Not applicable (0) |
| --- | --- | --- | --- | --- |
| 1. Was the study’s target population a close representation of the national population in relation to relevant variables, e.g. age, sex, occupation? |  |  |  |  |
| 2. Was the sampling frame a true or close representation of the target population? |  |  |  |  |
| 3. Was some form of random selection used to select the sample, OR was a census undertaken? |  |  |  |  |
| 4. Was the likelihood of non-response bias minimal? |  |  |  |  |
| 5. Were data collected directly from the subjects (as opposed to a proxy)? |  |  |  |  |
| 6. Was an acceptable case definition used in the study? |  |  |  |  |
| 7. Was the study instrument that measured the parameter of interest shown to have reliability and validity (if necessary)? |  |  |  |  |
| 8. Was the same mode of data collection used for all subjects? |  |  |  |  |
| 9. Was the length of the shortest prevalence period for the parameter of interest appropriate? |  |  |  |  |
| 10. Were the numerator(s) and denominator(s) for the parameter of interest appropriate? |  |  |  |  |
| Summary item on the overall risk of study bias |  |  |  |  |
| Interpretation of the risk of bias tool  • 7-10: Low risk of bias  • 4-6: Moderate risk of bias  • 0-3: High risk of bias | | | | |

Modified from: Hoy D, Brooks P, Woolf A, Blyth F, March L, Bain C, et al. Assessing risk of bias in prevalence studies: modification of an existing tool and evidence of interrater agreement. J Clin Epidemiol. 2012;65: 934–939. doi:10.1016/j.jclinepi.2011.11.014
