# Supplementary material for: A mapping review of methicillin-resistant Staphylococcus aureus proportions, genetic diversity, and antimicrobial resistance patterns in Cameroon
Source: PLoS One. 2023 Dec 22;18(12):e0296267. doi: 10.1371/journal.pone.0296267 (PMC10745167; doi:10.1371/journal.pone.0296267)
Supplement: S4 Table — (DOCX) [file pone.0296267.s004.docx]

S4 Table: Risk of bias assessment

| Author | Year (Year of publication) | Population categories | 1. Was the study’s target population a close representation of the national population in relation to relevant variables, e.g. age, sex, occupation? | 2. Was the sampling frame a true or close representation of the target population? | 3. Was some form of random selection used to select the sample, OR was a census undertaken? | 4. Was the likelihood of non-response bias minimal? | 5. Were data collected directly from the subjects (as opposed to a proxy)? | 6. Was an acceptable case definition used in the study? | 7. Was the study instrument that measured the parameter of interest shown to have reliability and validity (if necessary)? | 8. Was the same mode of data collection used for all subjects? | 9. Was the length of the shortest prevalence period for the parameter of interest appropriate (>1 year)? | 10. Were the numerator(s) and denominator(s) for the parameter of interest appropriate? | Score | Risk of bias |
| --- | --- | --- | --- | --- | --- | --- | --- | --- | --- | --- | --- | --- | --- | --- |
| Bissong | 2016 | Humans | No | Yes | No | No | Yes | Yes | Yes | Yes | No | Yes | 6 | Moderate risk of bias |
| Bissong | 2020 | Foods | No | Yes | Yes | Yes | Not applicable | Yes | Yes | Yes | No | Yes | 7 | Low risk of bias |
| Eyoh | 2013 | Humans | No | Yes | No | No | Yes | Yes | Yes | Yes | Unclear | Yes | 6 | Moderate risk of bias |
| Eyoh | 2021 | Humans | No | Yes | No | No | Yes | Yes | Yes | Yes | Yes | Yes | 7 | Low risk of bias |
| Foloum | 2021 | Humans | No | Yes | No | No | Yes | Yes | Yes | Yes | Yes | Yes | 7 | Low risk of bias |
| Founou | 2019 | Animals | No | Yes | No | No | Yes | Yes | Yes | Yes | No | Yes | 6 | Moderate risk of bias |
| Gonsu | 2013 | Humans | No | Yes | No | No | Yes | Yes | Yes | Yes | No | Yes | 6 | Moderate risk of bias |
| Gonsu | 2020 | Humans | No | Yes | No | No | Yes | Yes | Yes | Yes | No | Yes | 6 | Moderate risk of bias |
| Kengne | 2020 | Humans | No | Yes | No | No | Yes | Yes | Yes | Yes | No | Yes | 6 | Moderate risk of bias |
| Kengne | 2019 | Humans | No | Yes | No | No | Yes | Yes | Yes | Yes | Yes | Yes | 7 | Low risk of bias |
| Kesah | 2013 | Humans | No | Yes | No | No | Yes | Yes | Yes | Yes | No | Yes | 6 | Moderate risk of bias |
| Kesah | 2003 | Humans | No | Yes | No | No | Yes | Yes | Yes | Yes | Yes | Yes | 7 | Low risk of bias |
| Manhafo | 2021 | Humans | No | Yes | No | No | Yes | Yes | Yes | Yes | No | Yes | 6 | Moderate risk of bias |
| Marbou | 2020 | Humans | No | Yes | No | No | Yes | Yes | Yes | Yes | Yes | Yes | 7 | Low risk of bias |
| Massongo | 2021 | Humans | Yes | Yes | No | No | Yes | Yes | Yes | No | Yes | Yes | 7 | Low risk of bias |
| Mohamadou | 2022 | Humans | No | Yes | No | No | Yes | Unclear | Yes | Yes | Yes | Yes | 6 | Moderate risk of bias |
| Nankam | 2021 | Humans | No | Yes | No | No | Yes | Yes | Yes | Yes | No | Yes | 6 | Moderate risk of bias |
| Ngalani | 2020 | Humans | No | Yes | No | No | Yes | Yes | Yes | Yes | Yes | Yes | 7 | Low risk of bias |
| Njoungang | 2015 | Humans | No | Yes | No | No | Yes | Yes | Yes | Yes | No | Yes | 6 | Moderate risk of bias |
| Nkie Esemu | 2021 | Humans, Environment, Foods | No | Yes | Yes | No | Yes | Yes | Yes | Yes | No | Yes | 7 | Low risk of bias |
| Nkwelang | 2009 | Humans, Environment | No | Yes | No | No | Yes | Yes | Yes | Yes | Unclear | Yes | 6 | Moderate risk of bias |
| Sinda | 2020 | Humans | No | Yes | No | No | Yes | Yes | Yes | Yes | No | Yes | 6 | Moderate risk of bias |
| Straus | 2015 | Humans | No | No | Unclear | Unclear | Yes | Unclear | Yes | Unclear | Yes | Yes | 4 | Moderate risk of bias |
| Takemegni | 2021 | Environment | No | Yes | Yes | No | Not applicable | Yes | Yes | Yes | No | Yes | 6 | Moderate risk of bias |
