# Supplementary material for: A mapping review of methicillin-resistant Staphylococcus aureus proportions, genetic diversity, and antimicrobial resistance patterns in Cameroon
Source: PLoS One. 2023 Dec 22;18(12):e0296267. doi: 10.1371/journal.pone.0296267 (PMC10745167; doi:10.1371/journal.pone.0296267)
Supplement: S5 Table — (DOCX) [file pone.0296267.s005.docx]

S5 Table: Individual characteristics of included studies

| Authors | Setting | City/ Town | Study period | Age range | Population categories | MRSA identification assay | Sample types |
| --- | --- | --- | --- | --- | --- | --- | --- |
| Bissong et al., 2020 | Community-based | Bamenda, Buea, Kumbo | Apr/2018-Nov/2018 | Not applicable | Foods | Culture, PCR, Kirby-Bauer disk diffusion method | Unclear |
| Bissong et al., 2016 | Hospital-based | Douala | Mar/2016-Jun/2016 | Unclear | Humans | Culture, Methicillin resistance was assessed by determining the resistance profile of S. aureus isolates to oxacillin [5]. | Ear, Pus, Throat, Urine, Genital |
| Eyoh et al., 2013 | Hospital-based | Yaounde | Unclear | Adults | Humans | Culture, disc diffusion method | Nares |
| Eyoh et al., 2021 | Hospital-based | Yaounde | Jan/2016-Jan/2017 | Unclear | Humans | Culture, PCR, disk diffusion method | Nares |
| Foloum et al., 2021 | Hospital-based | Yaounde | Jan/2017-Dec/2019 | All ages | Humans | Culture, ROSCO DIAGNOSTICA, cefoxitin disc test, disc diffusion method | Uro-genital |
| Founou et al., 2019 | Community-based | Unclear | Mar/2016-Oct/2016 | Unclear | Animals | Culture, PCR, cefoxitin disc test | Unclear |
| Gonsu et al., 2013 | Hospital-based | Douala, Yaounde, Limbe | Jan/2011-Apr/2011 | Adults | Humans | Culture, disc diffusion method | Nares |
| Gonsu et al., 2020 | Hospital-based | Yaounde | Aug/2018-Mar/2019 | All ages | Humans | Culture, disc diffusion method | Nares |
| Kengne et al., 2020 | Hospital-based | Yaounde | Nov/2013-Mar/2014 | Adults | Humans | Culture, agar diffusion, cefoxitin disk | Pus |
| Kengne et al., 2019 | Hospital-based | Yaounde | Jan/2014-Nov/2016; Jun/2016-Nov/2016 | Unclear | Humans | Culture, Morphological characteristics; Biochemical examination; disk diffusion method | Blood, Ear, Pus, Throat, Urine, aspirates, urethral swabs, vaginal swabs and semen |
| Kesah et al., 2013 | Hospital-based | Dschang | May/2009-Mar/2010 | Unclear | Humans | Culture, E test | Ear, Pus, Urine, aspirates, swabs of wounds, burns, surgical site, eye, and skin |
| Kesah et al., 2003 | Hospital-based | Yaounde | 1996-1997 | Unclear | Humans | Culture, E test | Blood, Cerebrospinal fluid, Ear, Nares, Pus, Sputum, Throat, Urine, aspirates, swabs |
| Manhafo et al., 2021 | Hospital-based | Dschang | Jan/2021-May/2021 | All ages | Humans | Culture, CA-MRSA, disk diffusion method | Pus |
| Marbou et al., 2020 | Hospital-based | Mbouda | May/2016-May/2018 | Adults | Humans | Culture, PCR, (nuc gene); mecA gene; Kirby-Bauer disk diffusion method | Fecal |
| Massongo et al., 2021 | Hospital-based | Yaounde, other regions of the country | 2010 and 2017 | All ages | Humans | Culture, Vitek 2 Compact (Biomerieux) automaton and API kits; disk, e-test, liquid medium for Vitek 2 | Blood, Pus, Sputum, Stools, Urine, 7,314 patients (Bacteria positive) |
| Mohamadou et al., 2022 | Hospital-based | Unclear | Apr/2019-Dec/2020 | All ages | Humans | Culture, PCR, Kirby Bauer disc diffusion method | Blood cult, Pus, Semen, Stool, Surgery wound, Urethral, Urine, Vaginal cult |
| Nankam et al., 2021 | Hospital-based | Bangangte | Feb/2019-May/2019 | All ages | Humans | Culture, disk diffusion method, admission | Nares |
| Ngalani et al., 2020 | Hospital-based | Bafang | Nov/2016-Sep/2019 | Adults | Humans | Culture, disk diffusion method | Stools |
| Njoungang et al., 2015 | Hospital-based | Yaounde | Jun/2013-Dec/2013 | Unclear | Humans | Culture, Kirby- Bauer method | Ear, Pus, Urine, genital swabs, bone fragments |
| Nkie Esemu et al., 2021 | Community-based | Buea | Mar/2020-Aug/2020 | Adults | Humans, Environment, Foods | Culture, PCR, nuc and mecA genes; Kirby-Bauer disk diffusion method | Meat samples, butchery equipment (including knives, butchering slabs, and weighing balances), and hands of meat handlers |
| Nkwelang et al., 2009 | Hospital-based | Buea | Unclear | Unclear | Humans, Environment | Culture, Kirby-Bauer disk diffusion test | Swabs from wounds and health personnel (finger nails and nostrils), Environmental samples were formites, floors, benches, furniture (cupboards, beds), sinks, taps, switches, routine laboratory and surgical equipment. |
| Sinda et al., 2020 | Hospital-based | Buea, Yaounde, Limbe | Jan/2019-Jun/2019 | Adults | Humans | Culture, Kirby-Bauer method; nosocomial methicillin-resistant S. aureus | Nares |
| Straus et al., 2015 | Unclear | Unclear | 2005-2013 | Unclear | Humans | Whole genome sequencing | Unclear |
| Takemegni et al., 2021 | Hospital-based | Unclear | Dec/2018-May/2019 | Not applicable | Environment | Culture, Kirby–Bauer disc-diffusion method | Not applicable |
