# Supplementary material for: A mapping review of methicillin-resistant Staphylococcus aureus proportions, genetic diversity, and antimicrobial resistance patterns in Cameroon
Source: PLoS One. 2023 Dec 22;18(12):e0296267. doi: 10.1371/journal.pone.0296267 (PMC10745167; doi:10.1371/journal.pone.0296267)
Supplement: S6 Table — (DOCX) [file pone.0296267.s006.docx]

S6 Table: Individual characteristics of MRSA prevalence studies in humans

| Author | Setting | Setting | Setting | Regions | City/ Town | Study period | Age range | Population sub-category | definition of conditions | MRSA identification assay | Sample types | Estimates | No. of participants | Total no. of isolates | No. of S. aureus positives | No. of MRSA positives | Prevalence |
| --- | --- | --- | --- | --- | --- | --- | --- | --- | --- | --- | --- | --- | --- | --- | --- | --- | --- |
| Eyoh et al., 2021 | Hospital-based | Out-patients | Urban | Centre | Yaounde | Jan/2016-Jan/2017 | Unclear | Healthcare workers (S aureus isolates) | Colonization/ carriage | Culture, PCR, disk diffusion method | Nares | Centre |  |  | 20 | 9 | 45,0 |
| Eyoh et al., 2021 | Hospital-based | Out-patients | Urban | Centre | Yaounde | Jan/2016-Jan/2017 | Unclear | Patients with diabetes mellitus (S aureus isolates) | Colonization/ carriage | Culture, PCR, disk diffusion method | Nares | Centre |  |  | 18 | 7 | 38,9 |
| Eyoh et al., 2021 | Hospital-based | Out-patients | Urban | Centre | Yaounde | Jan/2016-Jan/2017 | Unclear | Patients with HIV/AIDS (S aureus isolates) | Colonization/ carriage | Culture, PCR, disk diffusion method | Nares | Centre |  |  | 57 | 19 | 33,3 |
| Mohamadou et al., 2022 | Hospital-based |  | Unclear | Adamawa | Unclear | Apr/2019-Dec/2020 | All ages | Patients with various diseases (S aureus isolates) | Unclear | Culture, PCR, Kirby Bauer disc diffusion method | Blood cult, Pus, Semen, Stool, Surgery wound, Urethral, Urine, Vaginal cult | Adamawa |  |  | 201 | 52 | 25,9 |
| Mohamadou et al., 2022 | Hospital-based |  | Unclear | Far North | Unclear | Apr/2019-Dec/2020 | All ages | Patients with various diseases (S aureus isolates) | Unclear | Culture, PCR, Kirby Bauer disc diffusion method | Blood cult, Pus, Semen, Stool, Surgery wound, Urethral, Urine, Vaginal cult | Far North |  |  | 179 | 40 | 22,3 |
| Bissong et al., 2016 | Hospital-based | In and out patients | Urban | Littoral | Douala | Mar/2016-Jun/2016 | Unclear | Patients with skin and soft tissue infections | Unclear | Culture, Methicillin resistance was assessed by determining the resistance profile of S. aureus isolates to oxacillin [5]. | Ear, Pus, Throat, Urine, Genital | Littoral | 114 |  |  | 15 | 13,2 |
| Esemu et al., 2021 | Community-based |  | Urban | South West | Buea | Mar/2020-Aug/2020 | Adults | Asymptomatic patients | Colonization/ carriage | Culture, PCR, nuc and mecA genes; Kirby-Bauer disk diffusion method | Meat samples, butchery equipment (including knives, butchering slabs, and weighing balances), and hands of meat handlers | South West | 52 |  |  | 3 | 5,8 |
| Eyoh et al., 2013 | Hospital-based |  | Urban | Centre | Yaounde | Unclear | Adults | Healthcare workers | Colonization/ carriage | Culture, disc diffusion method | Nares | Centre | 254 |  |  | 8 | 3,1 |
| Foloum et al., 2021 | Hospital-based | Internal medicine, obstetrics and gynecology, pediatrics and child health and general medicine and one laboratory | Urban | Centre | Yaounde | Jan/2017-Dec/2019 | All ages | Clinically ill and asymptomatic patients | Clinical infection, Colonization/ carriage | Culture, ROSCO DIAGNOSTICA, cefoxitin disc test, disc diffusion method | Uro-genital | Centre | 1683 |  |  | 47 | 2,8 |
| Gonsu et al., 2013 | Hospital-based | In-patients | Urban | Multiregions | Douala, Yaounde, Limbe | Jan/2011-Apr/2011 | Adults | Healthcare workers; Patients with various diseases | Colonization/ carriage | Culture, disc diffusion method | Nares | Multiregions | 295 |  |  | 102 | 34,6 |
| Gonsu et al., 2020 | Hospital-based | Intensive Care Units | Urban | Centre | Yaounde | Aug/2018-Mar/2019 | All ages | Patients with various diseases | Colonization/ carriage | Culture, disc diffusion method | Nares | Centre | 127 |  |  | 17 | 13,4 |
| Kengne et al., 2020 | Hospital-based |  | Urban | Centre | Yaounde | Nov/2013-Mar/2014 | Adults | Patients with HIV/AIDS | Unclear | Culture, agar diffusion, cefoxitin disk | Pus | Centre | 53 |  |  | 1 | 1,9 |
| Kesah et al., 2013 | Hospital-based |  | Rural | West | Dschang | May/2009-Mar/2010 | Unclear | Patients with various diseases (S aureus isolates) | Unclear | Culture, E test | Ear, Pus, Urine, aspirates, swabs of wounds, burns, surgical site, eye, and skin | West |  |  | 100 | 43 | 43,0 |
| Manhafo et al., 2021 | Hospital-based | In-patient (traumatology unit) | Rural | West | Dschang | Jan/2021-May/2021 | All ages | Patients with suppurating wounds or abscesses | Unclear | Culture, CA-MRSA, disk diffusion method | Pus | West | 52 |  |  | 9 | 17,3 |
| Marbou et al., 2020 | Hospital-based | Out-patients | Rural | West | Mbouda | May/2016-May/2018 | Adults | Clinically ill (Metabolic syndrome) and asymptomatic patients | Unclear | Culture, PCR, (nuc gene); mecA gene; Kirby-Bauer disk diffusion method | Fecal | West | 604 |  |  | 91 | 15,1 |
| Massongo et al., 2021 | Hospital-based |  | Mixed | Multiregions | National | 2010 and 2017 | All ages | Positive isolates | Unclear | Culture, Vitek 2 Compact (Biomerieux) automaton and API kits; disk, e-test, liquid medium for Vitek 2 | Blood, Pus, Sputum, Stools, Urine, 7,314 patients (Bacteria positive) | Multiregions |  | 10218 |  | 412 | 4,0 |
| Nankam et al., 2021 | Hospital-based | In-patients (surgical department) | Rural | West | Bangangte | Feb/2019-May/2019 | All ages | Patients with various diseases | Unclear | Culture, disk diffusion method, admission | Nares | West | 52 |  |  | 7 | 13,5 |
| Ngalani et al., 2020 | Hospital-based |  | Rural | West | Bafang | Nov/2016-Sep/2019 | Adults | Pregnant and non pregnant | Unclear | Culture, disk diffusion method | Stools | West | 129 |  |  | 53 | 41,1 |
| Njoungang et al., 2015 | Hospital-based | In and out patients | Urban | Centre | Yaounde | Jun/2013-Dec/2013 | Unclear | Unclear | Unclear | Culture, Kirby- Bauer method | Ear, Pus, Urine, genital swabs, bone fragments | Centre | 201 |  |  | 28 | 13,9 |
| Nkwelang et al., 2009 | Hospital-based |  | Urban | South West | Buea | Unclear | Unclear | Patients with wounds and health personnel (finger nails and nostrils) | Unclear | Culture, Kirby-Bauer disk diffusion test | Swabs from wounds and health personnel (finger nails and nostrils), Environmental samples were formites, floors, benches, furniture (cupboards, beds), sinks, taps, switches, routine laboratory and surgical equipment. | South West | 231 |  |  | 80 | 34,6 |
| Sinda et al., 2020 | Hospital-based | Surgery, intensive care unit, medicine and pediatric | Urban | Multiregions | Buea, Yaounde, Limbe | Jan/2019-Jun/2019 | Adults | Patients with various diseases | Colonization/ carriage | Culture, Kirby-Bauer method; nosocomial methicillin-resistant S. aureus | Nares | Multiregions | 346 |  |  | 162 | 46,8 |
