# Supplementary material for: A mapping review of methicillin-resistant Staphylococcus aureus proportions, genetic diversity, and antimicrobial resistance patterns in Cameroon
Source: PLoS One. 2023 Dec 22;18(12):e0296267. doi: 10.1371/journal.pone.0296267 (PMC10745167; doi:10.1371/journal.pone.0296267)
Supplement: S7 Table — (DOCX) [file pone.0296267.s007.docx]

S7 Table: Individual characteristics of MRSA prevalence studiesin animals, foods, and environment

| Author | Main setting | Specific location | Geographical classification | City/ Town | Study period | Age range | Population categories | Population sub-category | MRSA identification assay | No. of participants | No. of MRSA positives |
| --- | --- | --- | --- | --- | --- | --- | --- | --- | --- | --- | --- |
| Bissong et al., 2020 | Community-based | Abbatoirs | Mixed | Bamenda, Buea, Kumbo | Apr/2018-Nov/2018 | Not applicable | Foods | Milk and Beef | Culture, PCR, Kirby-Bauer disk diffusion method | 250 | 8 |
| Founou et al., 2019 | Community-based | three slaughterhouses/markets | Unclear | Unclear | Mar/2016-Oct/2016 | Unclear | Animals | Pigs | Culture, PCR, cefoxitin disc test | 432 | 1 |
| Nkie Esemu et al., 2021 | Community-based | Not specified | Urban | Buea | Mar/2020-Aug/2020 | Adults | Environment | Knives | Culture, PCR, nuc and mecA genes; Kirby-Bauer disk diffusion method | 52 | 11 |
| Nkie Esemu et al., 2021 | Community-based | Not specified | Urban | Buea | Mar/2020-Aug/2020 | Adults | Environment | Weighing balances | Culture, PCR, nuc and mecA genes; Kirby-Bauer disk diffusion method | 52 | 0 |
| Nkie Esemu et al., 2021 | Community-based | Not specified | Urban | Buea | Mar/2020-Aug/2020 | Adults | Environment | Butchering slabs | Culture, PCR, nuc and mecA genes; Kirby-Bauer disk diffusion method | 52 | 5 |
| Nkie Esemu et al., 2021 | Community-based | Not specified | Urban | Buea | Mar/2020-Aug/2020 | Adults | Foods | Meat | Culture, PCR, nuc and mecA genes; Kirby-Bauer disk diffusion method | 52 | 8 |
| Nkwelang et al., 2009 | Hospital-based | Not specified | Urban | Buea | Unclear | Unclear | Environment | Formites, floors, benches, furniture (cupboards, beds), sinks, taps, switches, routine laboratory and surgical equipment | Culture, Kirby-Bauer disk diffusion test | 231 | 80 |
| Takemegni et al., 2021 | Hospital-based | Medical, Paediatric, Operating Theatre, Laboratory, Surgical, Emergency and Maternity | Unclear | Unclear | Dec/2018-May/2019 | Not applicable | Environment | equipment and materials | Culture, Kirby–Bauer disc-diffusion method | 89 | 30 |
