# Supplementary material for: A mapping review of methicillin-resistant Staphylococcus aureus proportions, genetic diversity, and antimicrobial resistance patterns in Cameroon
Source: PLoS One. 2023 Dec 22;18(12):e0296267. doi: 10.1371/journal.pone.0296267 (PMC10745167; doi:10.1371/journal.pone.0296267)
Supplement: S8 Table — (DOCX) [file pone.0296267.s008.docx]

S8 Table: Antimicrobial resistance patterns of MRSA isolates

| Author | City/ Town | Study period | Population sub-category | Antibiotic resistance profile of MRSA isolates : Antibiotic | No. of colonized participants with MRSA | N° of MRSA antibiotic-resistant isolates. | Antimicrobial resistance rates |
| --- | --- | --- | --- | --- | --- | --- | --- |
| Bissong et al., 2016 | Douala | Mar/2016-Jun/2016 | Patients with specific diseases | Amikacin | 15 | 0 | 0,0 |
| Bissong et al., 2016 | Douala | Mar/2016-Jun/2016 | Patients with specific diseases | Amoxicillin | 15 | 6 | 40,0 |
| Bissong et al., 2016 | Douala | Mar/2016-Jun/2016 | Patients with specific diseases | Erythromycin | 15 | 10 | 66,7 |
| Bissong et al., 2016 | Douala | Mar/2016-Jun/2016 | Patients with specific diseases | Gentamicin | 15 | 7 | 46,7 |
| Bissong et al., 2016 | Douala | Mar/2016-Jun/2016 | Patients with specific diseases | Lincomycin | 15 | 0 | 0,0 |
| Bissong et al., 2016 | Douala | Mar/2016-Jun/2016 | Patients with specific diseases | Ofloxacin | 15 | 0 | 0,0 |
| Bissong et al., 2016 | Douala | Mar/2016-Jun/2016 | Patients with specific diseases | oxacillin | 15 | 15 | 100,0 |
| Bissong et al., 2016 | Douala | Mar/2016-Jun/2016 | Patients with specific diseases | Penicillin | 15 | 12 | 80,0 |
| Bissong et al., 2016 | Douala | Mar/2016-Jun/2016 | Patients with specific diseases | Vancomycin | 15 | 1 | 6,7 |
| Kengne et al., 2019 | Yaounde | Jan/2014-Nov/2016; Jun/2016-Nov/2016 | Patients with specific diseases | Cefoxitin | 201 | 201 | 100,0 |
| Kengne et al., 2019 | Yaounde | Jan/2014-Nov/2016; Jun/2016-Nov/2016 | Patients with specific diseases | Chloramphnicol | 192 | 70 | 36,5 |
| Kengne et al., 2019 | Yaounde | Jan/2014-Nov/2016; Jun/2016-Nov/2016 | Patients with specific diseases | Ciprofloxacin | 199 | 53 | 26,6 |
| Kengne et al., 2019 | Yaounde | Jan/2014-Nov/2016; Jun/2016-Nov/2016 | Patients with specific diseases | Co-trimoxazole | 200 | 178 | 89,0 |
| Kengne et al., 2019 | Yaounde | Jan/2014-Nov/2016; Jun/2016-Nov/2016 | Patients with specific diseases | Doxycycline | 197 | 134 | 68,0 |
| Kengne et al., 2019 | Yaounde | Jan/2014-Nov/2016; Jun/2016-Nov/2016 | Patients with specific diseases | Erithromycin | 199 | 111 | 55,8 |
| Kengne et al., 2019 | Yaounde | Jan/2014-Nov/2016; Jun/2016-Nov/2016 | Patients with specific diseases | fusidic acid | 198 | 86 | 43,4 |
| Kengne et al., 2019 | Yaounde | Jan/2014-Nov/2016; Jun/2016-Nov/2016 | Patients with specific diseases | Gentamicin | 201 | 89 | 44,3 |
| Kengne et al., 2019 | Yaounde | Jan/2014-Nov/2016; Jun/2016-Nov/2016 | Patients with specific diseases | Kanamycin | 195 | 136 | 69,7 |
| Kengne et al., 2019 | Yaounde | Jan/2014-Nov/2016; Jun/2016-Nov/2016 | Patients with specific diseases | Lincomycin | 185 | 130 | 70,3 |
| Kengne et al., 2019 | Yaounde | Jan/2014-Nov/2016; Jun/2016-Nov/2016 | Patients with specific diseases | Pristinamycin | 195 | 74 | 37,9 |
| Kengne et al., 2019 | Yaounde | Jan/2014-Nov/2016; Jun/2016-Nov/2016 | Patients with specific diseases | Rifampicin | 201 | 31 | 15,4 |
| Kengne et al., 2019 | Yaounde | Jan/2014-Nov/2016; Jun/2016-Nov/2016 | Patients with specific diseases | Tobramycin | 189 | 137 | 72,5 |
| Kengne et al., 2019 | Yaounde | Jan/2014-Nov/2016; Jun/2016-Nov/2016 | Patients with specific diseases | Vancomycin | 197 | 157 | 79,7 |
| Kesah et al., 2003 | Yaounde | 1996-1997 | Patients with specific diseases | Ciprofloxacin | 27 | 23 | 85,2 |
| Kesah et al., 2003 | Yaounde | 1996-1997 | Patients with specific diseases | erythromycin | 27 | 12 | 44,4 |
| Kesah et al., 2003 | Yaounde | 1996-1997 | Patients with specific diseases | fusidic acid | 27 | 0 | 0,0 |
| Kesah et al., 2003 | Yaounde | 1996-1997 | Patients with specific diseases | gentamicin | 27 | 13 | 48,1 |
| Kesah et al., 2003 | Yaounde | 1996-1997 | Patients with specific diseases | Rifampin | 27 | 24 | 88,9 |
| Kesah et al., 2003 | Yaounde | 1996-1997 | Patients with specific diseases | Trimethoprim–sulfamethoxazole | 27 | 13 | 48,1 |
| Kesah et al., 2003 | Yaounde | 1996-1997 | Patients with specific diseases | vancomycin | 27 | 27 | 100,0 |
| Manhafo et al., 2021 | Dschang | Jan/2021-May/2021 | Patients with specific diseases | Amoxicillin | 9 | 7 | 77,8 |
| Manhafo et al., 2021 | Dschang | Jan/2021-May/2021 | Patients with specific diseases | Clindamycin | 9 | 2 | 22,2 |
| Manhafo et al., 2021 | Dschang | Jan/2021-May/2021 | Patients with specific diseases | erythromycin | 9 | 5 | 55,6 |
| Manhafo et al., 2021 | Dschang | Jan/2021-May/2021 | Patients with specific diseases | gentamicin | 9 | 2 | 22,2 |
| Manhafo et al., 2021 | Dschang | Jan/2021-May/2021 | Patients with specific diseases | oxacillin | 9 | 1 | 11,1 |
| Manhafo et al., 2021 | Dschang | Jan/2021-May/2021 | Patients with specific diseases | Penicillin | 9 | 4 | 44,4 |
| Manhafo et al., 2021 | Dschang | Jan/2021-May/2021 | Patients with specific diseases | vancomycin | 9 | 4 | 44,4 |
| Mohamadou et al., 2022 | Unclear | Apr/2019-Dec/2020 | Unclear | Amoxicillin-clavulanate | 92 | 43 | 46,7 |
| Mohamadou et al., 2022 | Unclear | Apr/2019-Dec/2020 | Unclear | Cefoxitin | 92 | 92 | 100,0 |
| Mohamadou et al., 2022 | Unclear | Apr/2019-Dec/2020 | Unclear | Ciprofloxacin | 92 | 39 | 42,4 |
| Mohamadou et al., 2022 | Unclear | Apr/2019-Dec/2020 | Unclear | Co-trimoxazole | 92 | 78 | 84,8 |
| Mohamadou et al., 2022 | Unclear | Apr/2019-Dec/2020 | Unclear | Erythromycin | 92 | 40 | 43,5 |
| Mohamadou et al., 2022 | Unclear | Apr/2019-Dec/2020 | Unclear | Fusidic acid | 92 | 7 | 7,6 |
| Mohamadou et al., 2022 | Unclear | Apr/2019-Dec/2020 | Unclear | Gentamicin | 92 | 51 | 55,4 |
| Mohamadou et al., 2022 | Unclear | Apr/2019-Dec/2020 | Unclear | Lincomycin | 92 | 17 | 18,5 |
| Mohamadou et al., 2022 | Unclear | Apr/2019-Dec/2020 | Unclear | Minocycline | 92 | 8 | 8,7 |
| Mohamadou et al., 2022 | Unclear | Apr/2019-Dec/2020 | Unclear | Ofloxacin | 92 | 63 | 68,5 |
| Mohamadou et al., 2022 | Unclear | Apr/2019-Dec/2020 | Unclear | Oxacillin | 92 | 92 | 100,0 |
| Mohamadou et al., 2022 | Unclear | Apr/2019-Dec/2020 | Unclear | Penicillin | 92 | 82 | 89,1 |
| Mohamadou et al., 2022 | Unclear | Apr/2019-Dec/2020 | Unclear | Pirlimycin | 92 | 3 | 3,3 |
| Mohamadou et al., 2022 | Unclear | Apr/2019-Dec/2020 | Unclear | Rifampicin | 92 | 7 | 7,6 |
| Mohamadou et al., 2022 | Unclear | Apr/2019-Dec/2020 | Unclear | Tetracycline | 92 | 65 | 70,7 |
| Mohamadou et al., 2022 | Unclear | Apr/2019-Dec/2020 | Unclear | Vancomycin | 92 | 12 | 13,0 |
| Njoungang et al., 2015 | Yaounde | Jun/2013-Dec/2013 | Unclear | Amikacin | 28 | 12 | 42,9 |
| Njoungang et al., 2015 | Yaounde | Jun/2013-Dec/2013 | Unclear | Cefoxitin | 28 | 28 | 100,0 |
| Njoungang et al., 2015 | Yaounde | Jun/2013-Dec/2013 | Unclear | Co-trimoxazole | 28 | 10 | 35,7 |
| Njoungang et al., 2015 | Yaounde | Jun/2013-Dec/2013 | Unclear | Doxycycline | 28 | 17 | 60,7 |
| Njoungang et al., 2015 | Yaounde | Jun/2013-Dec/2013 | Unclear | Erythromycin | 28 | 14 | 50,0 |
| Njoungang et al., 2015 | Yaounde | Jun/2013-Dec/2013 | Unclear | Gentamicin | 28 | 9 | 32,1 |
| Njoungang et al., 2015 | Yaounde | Jun/2013-Dec/2013 | Unclear | Lincomycine | 28 | 11 | 39,3 |
| Njoungang et al., 2015 | Yaounde | Jun/2013-Dec/2013 | Unclear | Netilmycine | 28 | 9 | 32,1 |
| Njoungang et al., 2015 | Yaounde | Jun/2013-Dec/2013 | Unclear | Ofloxacin | 28 | 14 | 50,0 |
| Njoungang et al., 2015 | Yaounde | Jun/2013-Dec/2013 | Unclear | Pefloxacin | 28 | 14 | 50,0 |
| Njoungang et al., 2015 | Yaounde | Jun/2013-Dec/2013 | Unclear | Penicillin | 28 | 28 | 100,0 |
| Njoungang et al., 2015 | Yaounde | Jun/2013-Dec/2013 | Unclear | Pristinamycine | 28 | 0 | 0,0 |
| Njoungang et al., 2015 | Yaounde | Jun/2013-Dec/2013 | Unclear | Tobramycine | 28 | 12 | 42,9 |
| Njoungang et al., 2015 | Yaounde | Jun/2013-Dec/2013 | Unclear | Vancomycin | 28 | 0 | 0,0 |
| Nkie Esemu et al., 2021 | Buea | Mar/2020-Aug/2020 | Asymptomatic patients, Knives , Weighing balances , Butchering slabs , Meat | Amikacin | 27 | 0 | 0,0 |
| Nkie Esemu et al., 2021 | Buea | Mar/2020-Aug/2020 | Asymptomatic patients, Knives , Weighing balances , Butchering slabs , Meat | Ampicillin | 27 | 27 | 100,0 |
| Nkie Esemu et al., 2021 | Buea | Mar/2020-Aug/2020 | Asymptomatic patients, Knives , Weighing balances , Butchering slabs , Meat | Azithromycin | 27 | 5 | 18,5 |
| Nkie Esemu et al., 2021 | Buea | Mar/2020-Aug/2020 | Asymptomatic patients, Knives , Weighing balances , Butchering slabs , Meat | Cefepime | 27 | 27 | 100,0 |
| Nkie Esemu et al., 2021 | Buea | Mar/2020-Aug/2020 | Asymptomatic patients, Knives , Weighing balances , Butchering slabs , Meat | Ciprofloxacin | 27 | 22 | 81,5 |
| Nkie Esemu et al., 2021 | Buea | Mar/2020-Aug/2020 | Asymptomatic patients, Knives , Weighing balances , Butchering slabs , Meat | Doxycycline | 27 | 5 | 18,5 |
| Nkie Esemu et al., 2021 | Buea | Mar/2020-Aug/2020 | Asymptomatic patients, Knives , Weighing balances , Butchering slabs , Meat | Erythromycin | 27 | 15 | 55,6 |
| Nkie Esemu et al., 2021 | Buea | Mar/2020-Aug/2020 | Asymptomatic patients, Knives , Weighing balances , Butchering slabs , Meat | Gentamicin | 27 | 0 | 0,0 |
| Nkie Esemu et al., 2021 | Buea | Mar/2020-Aug/2020 | Asymptomatic patients, Knives , Weighing balances , Butchering slabs , Meat | Ofloxacin | 27 | 25 | 92,6 |
| Nkie Esemu et al., 2021 | Buea | Mar/2020-Aug/2020 | Asymptomatic patients, Knives , Weighing balances , Butchering slabs , Meat | Vancomycin | 27 | 10 | 37,0 |
| Sinda et al., 2020 | Buea, Yaounde, Limbe | Jan/2019-Jun/2019 | Patients with specific diseases | Amoxicillin | 263 | 161 | 61,2 |
| Sinda et al., 2020 | Buea, Yaounde, Limbe | Jan/2019-Jun/2019 | Patients with specific diseases | Ampicillin | 263 | 244 | 92,8 |
| Sinda et al., 2020 | Buea, Yaounde, Limbe | Jan/2019-Jun/2019 | Patients with specific diseases | Ceftriazone | 263 | 238 | 90,5 |
| Sinda et al., 2020 | Buea, Yaounde, Limbe | Jan/2019-Jun/2019 | Patients with specific diseases | Fusidic Acid | 263 | 46 | 17,5 |
| Sinda et al., 2020 | Buea, Yaounde, Limbe | Jan/2019-Jun/2019 | Patients with specific diseases | Minocycline | 263 | 9 | 3,4 |
| Sinda et al., 2020 | Buea, Yaounde, Limbe | Jan/2019-Jun/2019 | Patients with specific diseases | Penicillin | 263 | 263 | 100,0 |
| Sinda et al., 2020 | Buea, Yaounde, Limbe | Jan/2019-Jun/2019 | Patients with specific diseases | Rifampicin | 263 | 157 | 59,7 |
| Sinda et al., 2020 | Buea, Yaounde, Limbe | Jan/2019-Jun/2019 | Patients with specific diseases | Vancomycin | 263 | 4 | 1,5 |
