# Supplementary material for: A mapping review of methicillin-resistant Staphylococcus aureus proportions, genetic diversity, and antimicrobial resistance patterns in Cameroon
Source: PLoS One. 2023 Dec 22;18(12):e0296267. doi: 10.1371/journal.pone.0296267 (PMC10745167; doi:10.1371/journal.pone.0296267)
Supplement: S9 Table — (DOCX) [file pone.0296267.s009.docx]

S9 Table: Genetic diversity of MRSA isolates in Cameroon

| Author et al., Year of publication | City/ Town | Study period | Population sub-category | Sample types | Genotyping method | MRSA genetic types | No. of MRSA positive participants | N° of MRSA isolates with genetic types | Proportion of types |
| --- | --- | --- | --- | --- | --- | --- | --- | --- | --- |
| Eyoh et al., 2021 | Yaounde | Jan/2016-Jan/2017 | Healthcare workers, Diabetes mellitus patients, HIV-positive patients | Nares | Multiplex PCR | SCCMec types II | 35 | 2 | 5,7 |
| Eyoh et al., 2021 | Yaounde | Jan/2016-Jan/2017 | Healthcare workers | Nares | Multiplex PCR | SCCMec types IV | 9 | 5 | 55,6 |
| Eyoh et al., 2021 | Yaounde | Jan/2016-Jan/2017 | Diabetes mellitus patients | Nares | Multiplex PCR | SCCMec types IV | 7 | 5 | 71,4 |
| Eyoh et al., 2021 | Yaounde | Jan/2016-Jan/2017 | HIV-positive patients | Nares | Multiplex PCR | SCCMec types IV | 19 | 15 | 78,9 |
| Eyoh et al., 2021 | Yaounde | Jan/2016-Jan/2017 | Healthcare workers, Diabetes mellitus patients, HIV-positive patients | Nares | Multiplex PCR | SCCMec types V | 35 | 8 | 22,9 |
| Eyoh et al., 2021 | Yaounde | Jan/2016-Jan/2017 | Healthcare workers | Nares | Multiplex PCR | PVL | 9 | 7 | 77,8 |
| Eyoh et al., 2021 | Yaounde | Jan/2016-Jan/2017 | Diabetes mellitus patients | Nares | Multiplex PCR | PVL | 7 | 4 | 57,1 |
| Eyoh et al., 2021 | Yaounde | Jan/2016-Jan/2017 | HIV-positive patients | Nares | Multiplex PCR | PVL | 19 | 6 | 31,6 |
| Mohamadou et al., 2022 | Unclear | Apr/2019-Dec/2020 | Patients with various diseases | Urine | PCR | Not typable | 92 | 2 | 2,2 |
| Mohamadou et al., 2022 | Unclear | Apr/2019-Dec/2020 | Patients with various diseases | Blood cult, Pus, Semen, Stool, Surgery wound, Urethral, Urine, Vaginal cult | PCR | SCCMec types I | 92 | 22 | 23,9 |
| Mohamadou et al., 2022 | Unclear | Apr/2019-Dec/2020 | Patients with various diseases | Blood cult, Pus, Semen, Stool, Surgery wound, Urethral, Urine, Vaginal cult | PCR | SCCMec types II | 92 | 7 | 7,6 |
| Mohamadou et al., 2022 | Unclear | Apr/2019-Dec/2020 | Patients with various diseases | Blood cult, Pus, Semen, Stool, Surgery wound, Urethral, Urine, Vaginal cult | PCR | SCCMec types III | 92 | 13 | 14,1 |
| Mohamadou et al., 2022 | Unclear | Apr/2019-Dec/2020 | Patients with various diseases | Blood cult, Pus, Semen, Stool, Surgery wound, Urethral, Urine, Vaginal cult | PCR | SCCMec types IV | 92 | 27 | 29,3 |
| Mohamadou et al., 2022 | Unclear | Apr/2019-Dec/2020 | Patients with various diseases | Blood cult, Pus, Semen, Stool, Surgery wound, Urethral, Urine, Vaginal cult | PCR | SCCMec types V | 92 | 21 | 22,8 |
| Mohamadou et al., 2022 | Unclear | Apr/2019-Dec/2020 | Patients with various diseases | Blood cult, Pus, Semen, Stool, Surgery wound, Urethral, Urine, Vaginal cult | PCR | PVL | 90 | 49 | 54,4 |
| Founou et al., 2019 | Unclear | Mar/2016-Oct/2016 | Pigs |  | Whole genome sequencing | ST398 | 1 | 1 | 100,0 |
| Straus et al., 2015 | Unclear | 2005-2013 |  |  | Whole genome sequencing | USA300 | 1 | 1 | 100,0 |
